# Student midwives’ knowledge, skills and competency in relation to the active management of the third stage of labour: A correlational study

**DOI:** 10.4102/curationis.v43i1.2054

**Published:** 2020-06-15

**Authors:** Fungai Muzeya, Hester Julie

**Affiliations:** 1 Department of Nursing, Faculty of Community Health Sciences, University of the Western Cape, Cape Town, South Africa

**Keywords:** Competency, intrapartum care, knowledge, midwifery, active management of third stage of labour, objective structured clinical examination

## Abstract

**Background:**

Lesotho has been experiencing health challenges as indicated by its high maternal mortality ratio of 620 per 100 000 live births for the year 2010, which has been linked to its limited human resources.

**Objectives:**

The knowledge and skills of final-year student nurse-midwives related to the active management of the third stage of labour were determined.

**Method:**

A quantitative, descriptive survey design was used to conduct this study with 99 final-year midwifery students at four nursing schools in Lesotho using stratified sampling. The structured questionnaire collected data on the knowledge and self-reported competency. Subsequently, the controlled cord traction marks, extracted from the objective structured clinical examination (OSCE), were compared to the self-reported competency of these midwifery students using R software version 3.4.0.

**Results:**

The mean score for knowledge and the OSCE was 73.8% (*n* = 99) and 77.2% (*n* = 99), respectively. The majority of respondents (95.2%, *n* = 99) rated themselves highly in terms of the active management of the third stage of labour competency. There was no correlation between the self-reported competency and knowledge (*r* = 0.08, *p* = 0.4402), and self-reported competency and OSCE scores (*r* = −0.004, *p* = 0.01).

**Conclusion:**

The high mean scores for the knowledge and the OSCE indicate that the theoretical component of the curriculum on the active management of the third stage of labour was effective in equipping final-year midwifery students with knowledge and skills to carry out this competency.

## Introduction

The sub-Saharan African region has the highest burden of disease in the world, which includes the highest rates of human immunodeficiency virus (HIV) infections, maternal deaths and morbidity, with the lowest density of human resources compared with other regions (Kyei-Nimakoh, Carolan-Olah & McCann 2016:52). Lesotho is one of the countries located within the sub-Saharan region and therefore it is no surprise that the main drivers of the deteriorating health trends in Lesotho include HIV and acquired immunodeficiency syndrome (AIDS), limited access to essential maternal and preventive healthcare services, poor-quality services and lack of access to essential drugs (Ministry of Development Planning Lesotho 2014:17). Lesotho’s challenges in terms of the health of its population and limited human resources are reflected in the high maternal mortality ratio (MMR) of 487 per 100 000 live births for 2015 (World Health Organization [WHO] 2016). This rate is slightly lower than the reported 2013 MMR of 510 for sub-Saharan Africa but much higher than the worldwide MMR of 210 per 100 000 live births reported for 2013 (Kyei-Nimakoh et al. 2016:52). It is acknowledged that post-partum haemorrhage is an important contributor to the high worldwide MMR (Stanton et al. 2009:207). Likewise, the report on the 296 maternal deaths recorded in Lesotho between 2011 and 2016 indicated that obstetric haemorrhage and hypertension accounted for 93 (31%) and 82 (28%), respectively, of these deaths (Mashea et al. 2018:43). Yet, it is asserted that post-partum haemorrhage is one of the most preventable causes of maternal morbidity and mortality (Baldvinsdottir, Blomberg & Lilliecreutz 2018:2).

Post-partum haemorrhage is defined as blood loss of 500 millilitre (mL) or more within 24 h of birth (WHO 2012:3) and severe post-partum haemorrhage is defined as a blood loss of at least 1000 mL (Boltman-Binkowski 2018:10; Sentilhes et al. 2016:14). Post-partum haemorrhage is further classified into primary or secondary to indicate if the haemorrhage occurred within the first 24 h of birth or after this period (Boltman-Binkowski 2018:10). However, both types of post-partum haemorrhage can be prevented by ensuring that (1) pregnant women receive good antenatal care, (2) the third stage of labour is actively managed and (3) all retained products of conception are removed (Baldvinsdottir, Blomberg & Lilliecreutz 2018:2; Weeks 2014:202).

The active management of the third stage of labour is endorsed as a prophylactic intervention for both the prevention and management of post-partum haemorrhage (Finlayson et al. 2018:2; WHO 2012:4). This post-partum haemorrhage prophylactic intervention includes three elements: administration of an uterotonic drug, delaying cord clamping between 1 and 3 min, and controlled cord traction (WHO 2012:5). The primary reason for the administration of uterotonic drugs is to prevent post-partum haemorrhage (Finlayson et al. 2018:13). Hence, WHO recommends that 10 international units of oxytocin, as the preferred uterotonic, should be administered routinely according to their integrated management of pregnancy and childbirth guidelines (WHO 2015). Controlled cord traction is performed by placing one hand above the symphysis pubis to provide counter-traction while pulling on the cord with the other hand (Finlayson et al. 2018:13). However, the use of uterotonics is regarded as the gold standard for the prevention of post-partum haemorrhage, while controlled cord traction is recommended only for settings where skilled birth attendants are available (WHO 2012:5). Nevertheless, the World Health Organization (WHO) encourages that training and educational programmes for healthcare professionals should include all three elements of the active management of the third stage of labour.

## Background

A contributing factor to Lesotho’s slow progress towards achieving the set maternal health targets is associated with the suboptimal proportion of births delivered by skilled professionals. The term ‘skilled professional’ refers to (WHO 2004 as cited in WHO 2008):

[*A*]n accredited health professional – such as a midwife, doctor or nurse – who has been educated and trained to proficiency in the skills needed to manage normal (uncomplicated) pregnancies, childbirth and the immediate postnatal period, and in the identification, management and referral of complications in women and newborns. (p. 1)

In 2014, it was reported that there were only 0.5 doctors and 6.2 nurses and midwives per 10 000 of the population in Lesotho. This was below the WHO African region average of 2.4 and 10.9 per 10 000 of the population for doctors and nurses and midwives, respectively (Ministry of Development Planning Lesotho 2014:119). Hence, Lesotho has endorsed the WHO recommendation to upscale the training and education of healthcare professionals (WHO 2013:11). One of the objectives of the *National Strategic Development Plan of Lesotho 2012/2013 to 2016/2017* was to increase the quantity and quality of healthcare human resources (Ministry of Development Planning Lesotho 2014:119). It was estimated that 3272 nursing and midwifery trained professionals were required to meet the country’s health needs (Ministry of Health and Social Welfare 2011:9). It is thus acknowledged that nurses and midwives are crucial for reducing MMRs because they are the majority healthcare professionals group in Lesotho (Phafoli et al. 2018:225). Hence, upscaling strategies for nursing and midwifery focused on increasing the numbers and implementing curricula reforms to address the gaps identified in the nurse training programmes in Lesotho (Botma 2014:26; Stender et al. 2014:3). Consequently, the government worked collaboratively with development partners on strategies to improve nursing and midwifery education, which included the implementation of the competency-based curricula for midwifery in 2014.

As part of this commitment, competency-based education (CBE) has been introduced for the diploma in midwifery as a curricula reform strategy in Lesotho since 2014. The CBE approach requires the learner to be actively engaged in all aspects of acquiring the knowledge, skills and professional behaviours needed to demonstrate competence in a discipline (International Confederation of Midwives [ICM] 2012:5). It involves students making use of pre-developed study guides or workbooks that have various teaching and learning activities to encourage learner engagement. In addition, classroom learning activities are implemented concurrently with simulation and work-integrated learning activities to develop the required competencies. Competencies refer to the knowledge, attitudes and skills that midwifery students should have acquired after completion of the midwifery education programme.

One of the core competencies taught in the competency-based midwifery programme is the active management of the third stage of labour, which aims to improve the management of the third-stage labour. This is expected to reduce the occurrence or incidence of mortality and morbidity secondary to post-partum haemorrhage in Lesotho. However, the management of post-partum haemorrhage has not improved and therefore it was necessary to investigate this competency or lack thereof in student nurse-midwives as they will become the future midwives.

This study thus sought to measure the knowledge of the implementation of the active management of the third stage of labour and the skill of controlled cord traction of student nurse-midwives.

### Research method and design

The aim of this study was to determine the knowledge and skills of the final-year student nurse-midwives in relation to the implementation of the 2012 WHO guidelines on the prevention and management of post-partum haemorrhage in relation to the active management of the third stage of labour competency. The research objectives were to (1) determine the baseline knowledge of student nurse-midwives on the three steps recommended for the active management of the third stage of labour according to the WHO, (2) determine the baseline competence (before graduation) skills of student nurse-midwives in relation to the skill of controlled cord traction and (3) compare the student nurse-midwives’ self-reported levels of competency in the active management of the third stage of labour against their knowledge test and Objective Structured Clinical Examination (OSCE) scores. The OSCE scores were used as proxy for the skill component of controlled cord traction.

### Study design

A quantitative, descriptive research design was applied using a structured questionnaire to collect data on demographics, knowledge and self-reported competency of student nurse-midwives in relation to the active management of the third stage of labour.

### Setting

The research was conducted at four nurse training institutions of the Christian Health Association of Lesotho Nurse Training Institute (CHAL-NTI), namely, Paray School of Nursing, Roma College of Nursing, Scott College of Nursing and Maluti Adventist College. There are a total of six nurse training institutions in Lesotho. Five are offering the diploma in nursing and midwifery and one is offering a degree in nursing and midwifery. These include the four CHAL-NTI institutions, the Nursing Department of the National Health Training College and the National University of Lesotho. The researcher selected the four nurse training institutions of the CHAL-NTI because they have fully implemented the competency-based curriculum of the diploma midwifery, and the final examinations were standardised across the four institutions at the time of research.

### Study population and sampling strategy

The study population included all student nurse-midwives (*N* = 141) in their final year of midwifery at the four CHAL-NTI schools of nursing for the 2015–2016 academic year. The student nurse-midwives, who met the inclusion criteria, completed and returned 104 structured questionnaires to the researcher. The inclusion criteria were final-year midwifery students registered at these institutions, who have completed the theory and practice components of midwifery and have completed the final-year OSCE at the participating nurse training institutions. The sample in this study comprised students who willingly completed the questionnaires and signed the informed consent forms.

The OSCE scores of consenting students were accessed from the CHAL-NTI examinations office. A proportional stratified sampling was used to get proportional random samples from each school of nursing. Listings of student nurse-midwives were obtained from the four CHAL-NTI nurse training institutions. A random grid table was created using Microsoft Excel 2013, which was used to randomly sample 104 student nurse-midwives across the four nurse training institutions. The sample size was calculated to be 104 student nurse-midwives, using Epi Info^TM^ software version 7.1.5.2 Centres for Disease Control and Prevention, cdc.gov/epiinfo/index.htm and the following parameters: *N* = 141 (based on the information obtained from the schools of nursing); expected frequency 50%; confidence limits 5%; design effect 1.0; confidence level 95%; and cluster effect 4.

Each nurse training institution was treated as a stratum. Proportional sampling was used to determine the sample size from each stratum. The formula for calculating the sample size at each stratum was as follows: stratum size/size of strata × sample size (see Table 1).

**TABLE 1:** Sample size calculation.

Training institution	Number of students	Calculation	Sample size
1	47	47/141 × 104	35
2	35	35/141 × 104	26
3	30	30/141 × 104	22
4	29	29/141 × 104	21
**Total**	**141**	-	**104**

### Data collection

Questionnaires were physically distributed by the first author to the four CHAL-NTI nurse training institutions during May 2016. Data were collected from student nurse-midwives who were randomly chosen using a random grid table and who consented to filling the questionnaire. Student numbers were requested from the student to enable the extraction of the respondents’ controlled cord traction OSCE station marks. The controlled cord traction OSCE station was part of a 10-station nurse-midwifery practical examination. The marks obtained from the controlled cord traction OSCE station were used as a proxy to assess the skill component. The controlled cord traction OSCE station was scored using a pretested and peer-reviewed checklist by trained midwifery educators. Nurse-midwifery OSCE stations, resource lists and checklists were set by a committee of trained midwifery educators who are from the CHAL-NTI institutions. The stations, resources lists and checklists were then moderated by midwifery educators who are not part of the CHAL-NTI institutions and stored for examination use. These external moderators have been trained on moderation of OSCE stations. To assess the knowledge and perceived competency, a structured questionnaire was used.

### Data collection instrument

The structured questionnaire was developed on the basis of the WHO guidelines on the active management of the third stage of labour (WHO 2012:13). Content validity was established through a review of the questionnaire by two advanced midwifery educators with extensive midwifery experience. The questionnaire was also pretested at the researcher’s institution on five student nurse-midwives who did not participate in the study. This activity helped to determine how much time it would take to complete the questionnaire and to identify any aspects of the questionnaire which needed further clarification.

The questionnaire comprised three sections. Section A consisted of seven items that included, amongst others, questions on the respondent’s age, gender, school of nursing attended and entry level of education into the midwifery course. In addition, it included three questions to determine the number of deliveries the respondent observed and conducted, with and without supervision by qualified midwifery personnel.

Section B contained 13 objective type knowledge questions based on the WHO guidelines on the active management of the third stage of labour (WHO 2012:13). These included, amongst others, questions on the definition of the third stage of labour, drugs used during the active management of the third stage of labour, timing of drug administration and cord clamping, goal of the active management of the third stage of labour, defining post-partum haemorrhage, identifying signs that will signal that cord traction is indicated, identifying delayed third stage of labour and four questions on the technique of controlled cord traction.

Ten of the questions were multiple-choice items with one correct answer and three distractors and the other three were true/false items. Respondents were scored 1 for a correct answer and 0 for a wrong answer. The highest possible score for knowledge was 13 and the lowest possible score was 0.

Section C consisted of six self-rating questions where respondents would rate themselves on the ability to carry out selected skills related to the Active Management of the Third Stage of Labour (AMTSL). Respondents rated themselves on their ability to carry out certain procedures using a 4-point rating scale ranging from ‘I am competent = 4’, ‘Have some skill but need more practice = 3’, ‘Have little skill and I need a lot of practice = 2’, to ‘Have no skill = 1’. The possible total score for the active management of the third stage of labour-related skills thus ranged from 0 to 24. The OSCE pass score for the active management of the third stage of labour-related skills station, which made up 60% of the total possible score, was 15 out of the possible 24.

Data on students’ knowledge of the active management of the third stage of labour and their self-rated, perceived competence were collected in May 2016 by the researcher (first author) from the four schools of nursing.

### Data analysis

Identifiers (institution and student numbers) were inserted on the completed questionnaires to extract the respondents’ information from the OSCE data set. Data were coded and analysed using Microsoft Excel 2013 and R software version 3.4.0. R Foundation for Statistical Computing, https://cran.r-project.org/bin/windows/base/old/3.4.0/. Descriptive statistics, such as frequency counts, percentages, means, standard deviations (SD) and confidence intervals (CI), were used to analyse the variables. Pearson correlation coefficients were computed for the knowledge, OSCE and self-reported competency scores.

### Ethical consideration

Ethical clearance was obtained from the National Health Research Ethics Committee in Lesotho (Registration number ID65-2016). Institutional permission was obtained from the respective nursing schools. Access to collect data (questionnaire and OSCE data sets) from each school of nursing was also obtained through the management of each of the schools of nursing. The researcher obtained informed consent from the respondents. Respondents were informed about their rights to withdraw at any time during the study without any negative consequences. The respondents’ anonymity was ensured as no names were required to be entered into the questionnaires. Identifiers (institution and student numbers) on questionnaires were kept confidential and were only used to access the OSCE scores. The researchers treated the respondents’ information confidentially, as this was not shared with any other person. Only the researchers had access to the completed questionnaires and OSCE data set. Data were collected at a time that was agreed upon with students at each institution.

## Results

Ninety-nine questionnaires were completed by student nurse-midwives from the four schools of nursing (32, 21, 26 and 20 respondents, respectively) out of an envisaged 104 (95.2% response rate). Five potential respondents who had been randomly selected either declined to participate or were not on campus at the time of data collection. The respondents’ ages ranged from 21 to 48 years, with a mean of 26.1 years (SD = 5.9 years). Women made up the larger proportion of the respondents (80.8%, *n* = 80) and men constituted 19.2% (*n* = 19) of the respondents. Most of the respondents (84.5%, *n* = 84) reported their entry-level qualification into the midwifery year of study as a General Certificate of Education with a Diploma in Nursing (Table 2).

**TABLE 2:** Characteristics of respondents (*n* = 99).

Demographics	Descriptive statistics			
	*n*	%	Mean	SD
**Age in years**	-	-	26.14	5.88
**Gender**				
Males	19	19.2	-	-
Females	80	80.8	-	-
**Entry level**				
General certificate education and diploma nursing	84	84.5	-	-
Certificate nursing assistant and diploma in nursing	13	13.1	-	-
Degree in nursing	2	2	-	-
**Experience**				
Observed deliveries	-	-	13.4	7.92
Supervised deliveries	-	-	29.6	11.7
Unsupervised deliveries	-	-	10.1	11.75

SD, standard deviation.

The highest mean number of the deliveries conducted by students prior to the study were supervised deliveries (mean = 29.6). Table 3 illustrates the distribution of these deliveries by school.

**TABLE 3:** Mean numbers of observed, supervised and unsupervised deliveries for respective schools of nursing.

School of nursing	Observed deliveries	Supervised deliveries	Unsupervised deliveries
1	10.0	31.1	19.2
2	13.8	26.8	10.1
3	13.3	34.9	7.6
4	15.0	27.8	6.0

### Knowledge of the active management of the third stage of labour

Of all the 13 knowledge questions, there were three questions that more than 80% of the respondents answered correctly. These included questions on defining post-partum haemorrhage (97%, *n* = 96), identifying the most preferred drug for the active management of the third stage of labour (96%, *n* = 95) and timing of that drug administration (89%, *n* = 88). About 70% – 79% of the respondents answered four questions correctly, three of which asked about the technique of controlled cord traction (79.8%, *n* = 79; 77.8%, *n* = 77; and 77.8%, *n* = 77, respectively) and one was on the goal of the active management of the third stage of labour (74.8%, *n* = 74) (Table 3).

Of the 99 respondents, only 65.7% (*n* = 65) answered correctly to the question dealing with the definition of the third stage of labour. The following questions were answered correctly by less than 60% of the respondents: timing of cord clamping (59.6%, *n* = 56), signs for controlled cord traction (50.5%, *n* = 50), identification of delayed third stage of labour (49.5%, *n* = 49), technique on controlled cord traction (56.6%, *n* = 56) and one question on the active and physiological management of the third stage of labour (42.2%, *n* = 42) (as indicated in Table 4).

**TABLE 4:** Frequency distribution of respondents’ knowledge regarding the active management of the third stage of labour (*N* = 104, *n* = 99).

Questions	Correct		Incorrect		Missing/don’t know	
	*n*	%	*n*	%	*n*	%
Defining post-partum haemorrhage	96	97.0	3	3.0	0	0.0
Recommended drug for AMTSL	95	96.0	4	4.0	0	0.0
Timing of drug administration	88	89.0	10	10.1	1	1.0
Controlled cord traction	79	79.8	20	20.2	0	0.0
Controlled cord traction	77	77.8	20	20.2	2	20.0
Controlled cord traction	77	77.8	21	21.2	1	1.0
Goal of AMTSL	74	74.8	25	25.2	0	0.0
Definition of the third stage of labour	65	65.7	27	27.3	8	8.1
Timing of cord clamping	59	59.6	40	40.4	0	0.0
Signs of controlled cord traction	50	50.5	49	49.5	0	0.0
Delayed third stage of labour	49	49.5	49	49.5	1	1.0
Controlled cord traction	56	56.6	42	42.4	1	1.0
AMTSL and physiological management of the third stage of labour	42	42.4	53	53.5	4	4.0

AMTSL, Active Management of the Third Stage of Labour.

The mean of the overall knowledge score was 73.8% (*n* = 99, SD = 13.13, 95% CI: 71.2% – 76.4%).

### Self-reported competency

In relation to the respondents’ self-rating of their abilities in skills pertaining to the active management of the third stage of labour, the majority of respondents reported that they are competent in the following skills: checking the comfort of the mother during controlled cord traction (91.9%, *n* = 91), administering oxytocin (90.9%, *n* = 90) and assessing the placenta (86.9%, *n* = 86). A small number of respondents reported competence in carrying out the following skills: assessment of blood loss after childbirth (67.7%, *n* = 67), management of post-partum haemorrhage (66.7%, *n* = 66) and controlled cord traction (66.7%, *n* = 66). The mean of the overall self-reported competency was 95.2% (*n* = 99, SD = 6.5, 95% CI: 93.9% – 96.5%).

### Skills in controlled cord traction

Skills were measured by extracting the OSCE scores for the controlled cord traction procedure. The mean for the OSCE scores for the station on controlled cord traction was 77.2% (*n* = 99, SD = 14.4, 95% CI: 74.3% – 80.1%).

## Discussion

The results of this study corroborate with other studies on student nurses which indicate that young female adults make up the largest proportion of nursing students (Mugomeri et al. 2016:530; Yigzaw et al. 2015:133).

Optimum clinical exposure is required for student nurse-midwives if they are to attain the required competencies for qualified nurse-midwives, including the active management of the third stage of labour. The Lesotho Diploma in Midwifery curriculum requirements for observing deliveries and conducting deliveries under supervision are 10 and 35, respectively. This is lower than the ICM recommendation that the number of births a midwife must conduct under supervision prior to graduation is 50 (United Nations Fund for Population Activities [UNFPA] 2017:30). This number varies across many countries in East and Southern Africa, from 20 in Ethiopia to 70 in Uganda. South Africa expects a minimum of 15 births conducted under supervision for midwives prior to graduation (UNFPA 2017:30).

According to the findings of this study, the deliveries that were observed by students across the four schools met and even exceeded the Lesotho Midwifery curriculum requirements. At the time of data collection, the mean number of supervised deliveries conducted by students during training was 29.6. For unsupervised deliveries, the mean number was 10.12, with one school having a mean score as high as 19.2. The above results indicate a lack in the clinical accompaniment of students during work-integrated learning activities, which could be linked to a lack of preceptors or clinical instructors. This corroborates a study conducted in Ethiopia, which linked inadequacy in the learning environment to inadequate teaching staff (Yigzaw et al. 2015:133). The lack of opportunity for students to gain practical experience, including deliveries, difficulty retaining teaching staff and lack of equipment, amongst others, has been identified as one of the major challenges that impede the provision of quality midwifery education (UNFPA 2017:29). Furthermore, a study carried out in India on midwifery training schools revealed that students reported having less clinical experience related to births attended (Sharma et al. 2015:7).

In the individual knowledge questions, responses from students varied. The majority of students (96%, *n* = 96) had knowledge of drug administration pertaining to the active management of the third stage of labour. According to the WHO, administering uterotonic drugs such as oxytocin is the most important intervention in the active management of the third stage of labour (WHO 2012:1). However, only 59.6% of student nurse-midwives had knowledge of delayed cord clamping, which is the second most important step in the active management of the third stage of labour as recommended by the WHO. Delayed cord clamping by between 1 and 3 min after birth is recommended while initiating essential newborn care. Delayed cord clamping has been shown to have benefits including reducing risk of infants getting iron-deficiency anaemia, intravascular haemorrhage and hypotension (Gungorduk et al. 2018:191). Another important step in the active management of the third stage of labour is the controlled cord traction. This step, together with uterine massage, is described as optional; however, it only applies to settings where a skilled birth attendant is present (WHO 2012). Controlled cord traction has been shown to reduce the mean time of the third stage of labour, mean blood loss and risk of blood loss. In addition, it reduces the risk of having to manually remove placenta (Gungorduk et al. 2018:191; Hofmyer, Mshweshwe & Gulmezoglu 2017:2). Controlled cord traction involves applying gentle traction on the umbilical cord and, at the same time, applying uterine counter traction just above the symphysis pubis. A majority of respondents (77.8%, *n* = 99) had knowledge regarding the controlled cord traction procedure (Hofmyer et al. 2015).

The results of this study imply that the student nurse-midwives were not familiar with all the components of the active management of the third stage of labour. These findings are consistent with that of a South African study conducted on healthcare professionals, including doctors and midwives, which showed that only 62.1% of the respondents were familiar with the components of the active management of the third stage of labour (Benedict, Steinberg & Raunbenheimer 2016:156). A similar study with midwives in Nigeria further revealed that 66.7% of respondents had knowledge of the active management of the third stage of labour, but only 41% practised it and that the required procedures were not followed (Oyetunde & Nkwonta 2015:25).

The duration of the third stage of labour is estimated to be between 6 and 30 min (Gungorduk et al. 2018:188). Regarding delayed third stage of labour, 49 students (49.5%) did not have knowledge about the condition’s definition and could not recognise the condition. This means that students may not be able to notice conditions like placental retention on a mother who has given birth. The study also corroborates a study conducted on Ethiopian student midwives exiting public nursing schools. In this case, students lacked satisfactory performance scores on essential competencies for safe and effective practice (Yigzaw et al. 2015:136). Furthermore, a study on student nurse-midwives in India reported less confidence in recognising and managing complications during the antenatal and postnatal phases, including carrying out basic intrapartum skills amongst them (Sharma et al. 2015:7).

One-third of the respondents reported that they had little or some competency in certain procedures and required more practice in procedures such as assessment of blood loss, management of post-partum haemorrhage and controlled cord traction. Andrikopoulou and D’Alton (2019:11–12) reported that the assessment of blood loss is still a major challenge in the care of women in labour and delivery, resulting in over- or under-estimation. They recommend that the time spent attending to the patient to get a picture of how the patient has been faring longitudinally helps to reach accurate assessments. A study by Sharma et al. (2015:12) reported that students expressed low confidence in managing intrapartum and post-partum complications before they could refer them. The results of this study indicate a need to strengthen those areas identified in the midwifery curriculum. The mean performance of students in relation to the OSCE showed that they had the skills of controlled cord traction. However, the results related to the number of deliveries conducted without supervision indicate a lack in terms of affording students the clinical experience needed for them to attain the competency related to the active management of the third stage of labour, including the skill of controlled cord traction.

The results of the study furthermore show that the student nurse-midwives’ scores on self-reported level of competency in the active management of the third stage of labour were higher than both the knowledge test and OSCE scores. The mean score of self-reported level of competency was 95.2% against the mean scores of 73.8% and 77.2% for knowledge and OSCE, respectively. This might be in contradiction with the finding in this study that one-third of the respondents indicated that they required more practice in procedures including controlled cord traction. This finding reflects the disadvantage of using a self-report which is liable to response bias.

## Conclusion

The study results indicate that the curriculum component on the active management of the third stage of labour was, to some extent, effective in imparting knowledge and skills regarding the active management of the third stage of labour to final-year student nurse-midwives. However, the respondents lacked knowledge regarding delayed cord clamping as a component, signs of controlled cord traction, and the definition of third stage of labour and identification of the delayed third stage of labour. The respondents identified the need for more practice to gain competency in practices such as management of post-partum haemorrhage, estimation of blood loss after delivery and controlled cord traction.

### Recommendations

The findings of this study indicate that further studies should be carried out to determine the effect of the curriculum on all competencies for nurse-midwifery. Further studies should also be conducted to investigate other factors that might influence the implementation of the competency-based curriculum for midwifery, such as institutional support and nurse educator capacity, amongst others. There is a need to strengthen the curriculum component to ensure that student nurse-midwives have adequate procedural knowledge of the AMTSL.

### Limitations of the study

This study was conducted at schools of nursing under the CHAL-NTI. Hence, the findings of the study should not be generalised to include all final-year student nurse-midwives in Lesotho.
